# Pulmonary lymphoepithelioma-like carcinoma: A case report with emphasis on computed tomography findings

**DOI:** 10.1097/MD.0000000000024453

**Published:** 2021-02-05

**Authors:** Lin Zhou, Xin-Yi Liu, Ya He, Lu-ting Li, Shao-Jin Zhang

**Affiliations:** a Department of Radiology, Pidu District People's Hospital; b Department of Radiology, Shenzhen Children's Hospital, Shantou University Medical College; c Department of Respiratory and Critical Medicine, Sichuan Academy of Medical Sciences and Sichuan Provincial People's Hospital, Chengdu, Sichuan; d Department of Interventional Medicine, The Fifth Affiliated Hospital, SunYat-sen University, Guangdong, China.

**Keywords:** computed tomography, diagnosis, pulmonary lymphoepithelioma-like carcinoma

## Abstract

**Rationale:**

Pulmonary lymphoepithelioma-like carcinoma (PLELC) is a rare type of primary malignant lung tumor characterized by Epstein-Barr virus infection, with, to the authors’ knowledge, a total of only 500 reported cases during the past 30 years worldwide. Histologically, PLELC is similar to undifferentiated nasopharyngeal carcinoma and poorly differentiated squamous cell carcinoma. However, although PLELC accounts for <1% of all lung cancers, it has a better prognosis and is usually detected in non-smokers and individuals of Asian ancestry.

**Patient concerns:**

The patient presented with chest distress of no apparent cause, dizziness, headaches, and a feeling of disequilibrium without remission, as well as a pulmonary nodule incidentally detected on contrast-enhanced computed tomography (CT).

**Diagnosis:**

PLELC was confirmed histopathologically rather than on preoperative CT; nevertheless, CT findings still contributed to the diagnosis.

**Interventions:**

The patient underwent thoracoscopic wedge resection of the affected lung.

**Outcomes:**

The patient recovered after the lung nodule was completely removed, and was discharged. No evidence of recurrence or metastasis was found at the latest follow-up appointment 2 months after the operation.

**Lessons:**

PLELC is a rare bronchogenic carcinoma associated with lymphatic tissue with a favorable prognosis in most cases. With nonspecific clinical symptoms, specific radiological findings may facilitate an early diagnosis in some cases, followed by timely surgical intervention.

## 1 Introduction

Pulmonary lymphoepithelioma-like carcinoma (PLELC) is a rare primary lung cancer subtype associated with Epstein-Barr virus (EBV) infection and is histologically similar to undifferentiated nasopharyngeal carcinoma. Although first reported in 1987 by Begin et al,^[1]^ PLELC accounts for <1% of all lung cancers to date.^[2]^ PLELC is more prevalent in Asians and is more common in non-smokers.^[3]^ PLELC may be difficult to diagnose because it is similar to undifferentiated nasopharyngeal carcinoma and squamous cell carcinoma poorly differentiated by histology. However, it has a better prognosis than these and other subtypes of lung cancer. There are no specific clinical manifestations, although cough is the most common clinical complaint. Computed tomography (CT) can reveal specific features that are valuable in the early diagnosis of PLELC.

In the present study, we analyzed specific CT findings from a 56-year-old woman diagnosed with PLELC.

## 2 Case report

**Figure 1:**
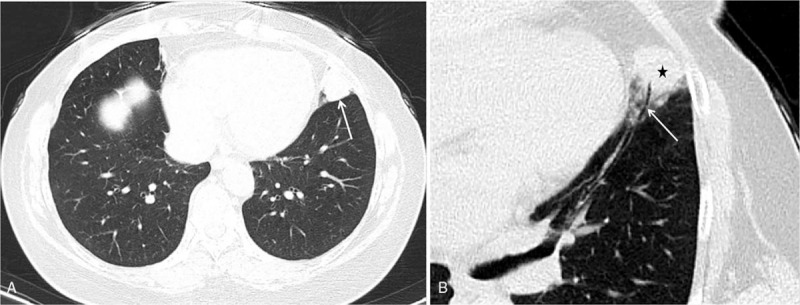
Unenhanced thoracic CT images in a 65-year-old woman with PLELC. The lung window shows a 2.1 × 1.8 cm peripheral nodule (A, arrow) in contact with the visceral pleura, exhibiting a positive lobulation sign (B, black star) and air bronchogram (B, arrow). CT = computed tomography, PLELC = pulmonary lymphoepithelioma-like carcinoma.

**Figure 2:**
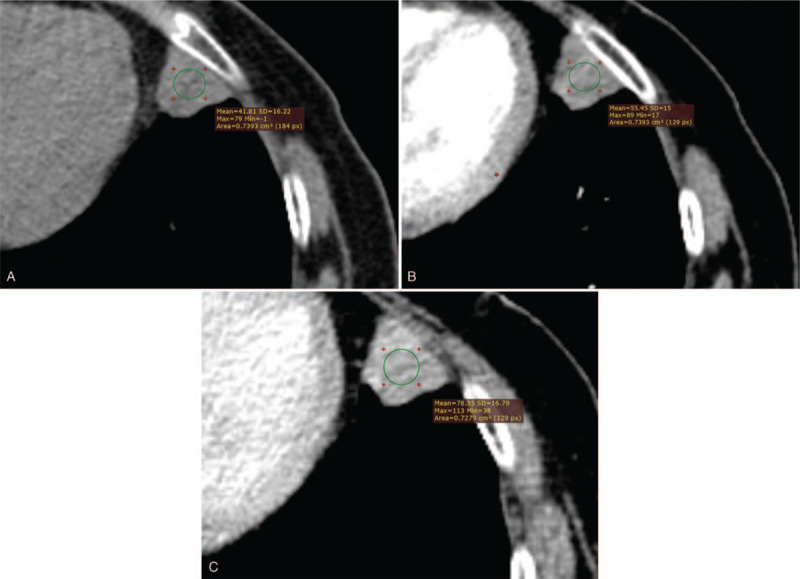
Enhanced thoracic CT images in a 65-year-old woman with PLELC. Soft-tissue window (A, B, C) shows obvious delayed enhancement of the nodule (red circle). CT = computed tomography, PLELC = pulmonary lymphoepithelioma-like carcinoma.

**Figure 3:**
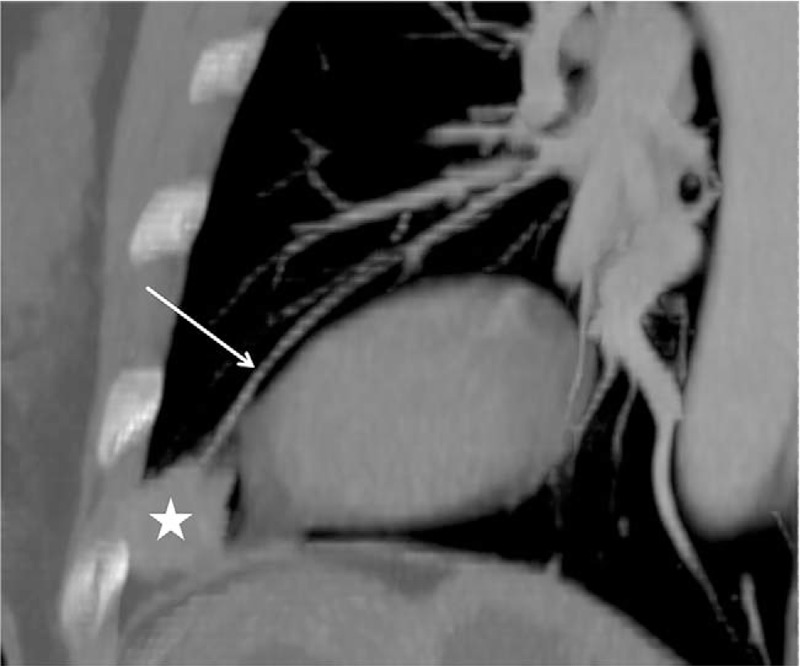
Oblique sagittal maximum intensity projection images in a 65-year-old woman with PLELC. The soft-tissue window shows the nodule (white star) and the closely related pulmonary veins (white arrow). PLELC = pulmonary lymphoepithelioma-like carcinoma.

**Figure 4:**
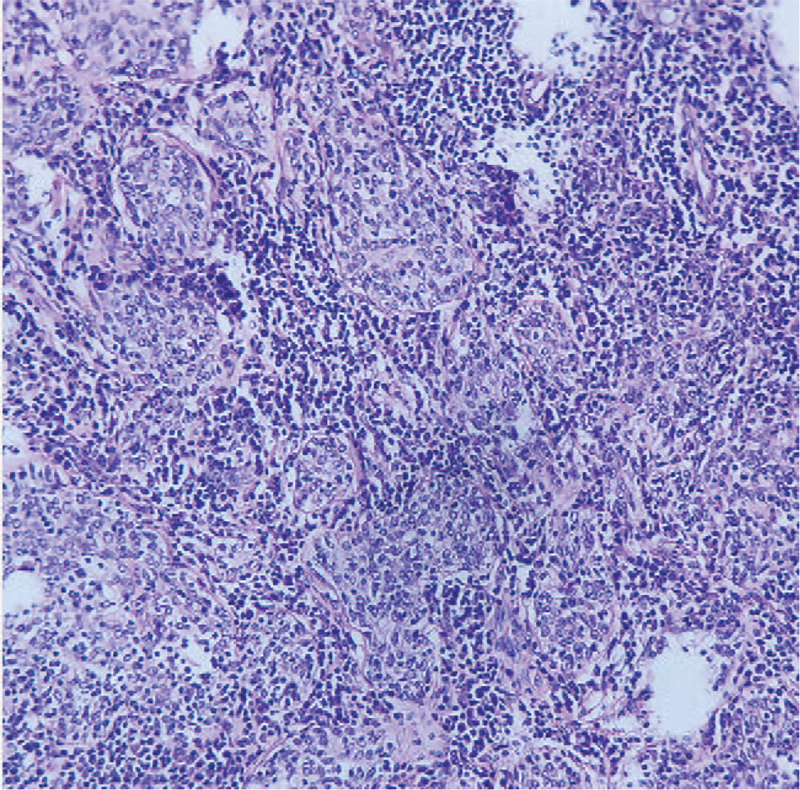
Low-power (20×) photomicrograph from a biopsy of PLELC depicting islands of malignant cells surrounded by lymphocytes and a population of plasma cells. PLELC = pulmonary lymphoepithelioma-like carcinoma.

The Institutional Review Board of Pidu District People's Hospital (Sichuan, China) approved this study. Written informed consent was obtained from the patient for treatment and publication of anonymized case details. A 56-year-old woman presented to hospital with complaints of chest distress that began 10 days earlier without any clearly defined inducing factors. She denied chest pain, compressing sensation in the precordium, or feelings that she was dying. However, she experienced dizziness and headaches, as well as a 4-month history of instability similar to stepping on cotton before visiting the hospital, which was not relieved by rest. There were no abnormalities detected on physical examination and laboratory tests, including peripheral blood check-up, and hepatic and renal functions. To rule out lung disease(s), the patient was referred to the department of radiology for chest CT, which was performed using a SOMATOM Definition AS+ spiral CT scanner (Siemens Medical Solutions, Forchheim, Germany) with a section thickness and slice interval of 8 mm. After the unenhanced section, the patient received intravenous bolus iohexol injection (100 mL) via the left median cubital vein at a rate of 4.5 mL/s. Axial images were captured from the thoracic inlet to the diaphragm, reformatted at 2 mm, and reconstructed using a soft-tissue and lung algorithm. Chest CT revealed a solid nodule with homogeneous density, measuring 2.1 × 1.8 cm in size, that was lodged in the peripheral region of the inferior lingual segment of the left upper lobe. The nodule exhibited a shallow lobulated appearance, with an irregular shape and obscured demarcations partially and closely attached to the adjacent pleura with an air bronchogram inside and an irregular shape with blurred boundaries attached to the visceral pleura (Fig. 1). Moreover, contrast-enhanced CT of the chest revealed obvious late-stage enhancement (Fig. 2) as well as closely related pulmonary veins (Fig. 3). No enlarged lymph nodes were found in the mediastinum or hilum of the lung. After multidisciplinary team consultation with the patient, she was referred to the department of thoracic surgery for thoracoscopic wedge resection of the affected lung. According to postoperative pathology results, the tumor cells gathered in piles of syncytial clusters and were arranged in a solid nest shape with large and vacuolar-shaped obvious nucleoli, rich cytoplasm, and abundant infiltration of lymphocytes and plasma cells in the surrounding stroma (Fig. 4). Immunohistochemistry results (P40 positive [+], CK+, CD20 [B cell+], p63+, TIF-1 negative [–], CD3 [T cell+], CK5/6+, Ki-67 [approximately 30% +], CD21 [FDC+], and EBER in situ hybridization +) contributed to confirming the diagnosis of PLELC.

After thoracoscopic wedge resection of the affected lung, enhanced CT confirmed complete removal of the pulmonary nodule, and no obvious enhanced nodules were detected. The patient was discharged from the hospital soon after completing pulmonary respiratory rehabilitation training. After 2 months of follow-up, the patient was informed of the outcome of her surgical therapy. In her view, heeding the doctor's advice and actively cooperating with the treatment plan was highly beneficial, considering her lack of expertise in this field. Presently, the patient is emotionally stable, with an optimistic attitude, and the latest check-up on enhanced CT (10 months after operation) revealed no evidence of recurrence or metastasis.

## 3 Discussion

Lymphoepithelioma-like carcinoma (LELC) is a clinically rare lymphoid-stroma-related tumor characterized by a low overall incidence of LELC^[3]^ and multiple potential primary sites of occurrence. To our knowledge, however, only 500 cases of PLELC— the most common LELC subtype—have been reported over the past 30 years. PLELC can occur at multiple sites including the lung(s), liver,^[4]^ breast,^[5]^ digestive tract,^[6]^ urinary tract,^[7]^ skin,^[8]^ thymus,^[3]^ and uterus.^[9]^ To date, it is widely accepted that the occurrence of PLELC is closely associated with EBV infection.^[10]^ PLELC patients experience non-specific clinical manifestations, and cough has been reported in most. Nearly one-quarter of these patients are asymptomatic, and some are diagnosed incidentally during routine physical examination.^[11]^ Chest CT^[12–14]^ often reveals peripheral nodules in direct contact with the adjacent pleura, with homogeneous attenuation, lobulation, and a spiculation sign,^[15]^ an air bronchus sign, few cavities and calcifications, no definite necrosis, and liquefaction.^[11]^ Enhanced CT reveals mild to moderate enhancement,^[13]^ and all CT scans usually reveal delayed enhancement,^[12]^ a vascular convergence sign, as well as invasion of the pleura and blood vessels.^[11]^ In the early stages, the bronchi are not invaded but the pleura usually is, which is mainly due to the fact that LECL originates from the interstitial lymphoid system of the lung. Thus, CT reveals that this tumor is generally proximal to the pleura and bronchi. By contrast, the tumor stroma of PLELC is rich in fibrous tissue, resulting in delayed enhancement on CT scan.

Regarding differential diagnosis, primary pulmonary lymphoma and other types of lung cancer must be considered. CT features of the former include ill-defined ground-glass nodules, pleural or chest wall involvement, and lymphatic or bronchovascular dissemination.^[16]^ Solitary subsolid or partly solid nodules with spiculation and pleural indentation for fibrosis indicate primary lung adenocarcinoma in the early stage(s), while solitary solid nodules or masses support a diagnosis of PLELC.^[17]^

PLELC is associated with few overt symptoms, and chest CT is the preferred tool for detection, not only because it is noninvasive but also because the focus and the nearby bronchovascular bundle can be revealed clearly without air or respiratory interference. PLELC should be considered in patients with non-obvious symptoms and CT characteristics such as peripheral solitary nodules, an air bronchus-charging sign, delayed enhancement, and few areas of necrosis.^[11]^ Additionally, the tissue structure of PLELC is consistent with that of primary nasopharyngeal LELC, thus making it exceedingly difficult to distinguish PLELC from pulmonary metastatic nasopharyngeal LELC on a histological basis alone. As a result, primary LELC of the nasopharynx must be excluded before making a diagnosis of PLELC.^[12]^ Ultimately, the confirmation of PLELC depends on pathology and immunohistochemical staining.

The prognosis of PLELC patients is often better that of patients with other types of lung cancer(s); the 5-year survival rate of patients with I/II stage PLELC can reach almost 100%.^[18]^ Some patients can survive for long periods after the initial operation, even those with subcutaneous metastases.^[19]^ Because most patients are diagnosed at an early stage, surgery remains an effective treatment option for most. PLELC has biological characteristics similar to undifferentiated nasopharyngeal carcinoma; therefore, it is speculated that it would exhibit equal sensitivity to radiotherapy as nasopharyngeal cancer.^[20]^ In fact, the tumor necrosis factor receptor associated factor 3 tumor suppressor gene is mutated in approximately 85% of PLELC (80% for deletions and 5% for other mutations); as such, it is believed that nuclear factor kappa-B inhibitors similar to tumor necrosis factor receptor associated factor 3 may be efficient chemotherapeutic drugs for PLELC patients.^[21]^ A study by Xie et al^[22]^ suggested that the high serological concentrations of EBV DNA in PLELC patients may be a biological marker with poor prognostic implications.

## 4 Conclusion

Although PLELC is a rare malignant tumor, it has a favorable prognosis. For a solitary peripheral pulmonary mass without obvious clinical symptoms, it is imperative to keep PLELC at the top of the list of differential diagnoses and actively dispense the necessary treatment after confirmation of the diagnosis on histology, provided that CT examination shows a lobulated appearance, a spicular sign, an air bronchus sign, no necrosis and cavitation, and exhibits delayed enhancement.

## Acknowledgments

Thanks are due to Zhi-fan Chen for assisting in preparation of this manuscript. Besides, they would like to thank Editage (www.editage.cn) for English language editing.

## Author contributions

**Conceptualization:** Lin Zhou, Xin-yi Liu, Shao-jin Zhang.

**Data curation:** Lin Zhou, Xin-yi Liu, Shao-jin Zhang.

**Formal analysis:** Lin Zhou, Xin-yi Liu, Lu-ting Li, Shao-jin Zhang.

**Funding acquisition:** Lin Zhou, Xin-yi Liu, Ya He, Shao-jin Zhang.

**Investigation:** Lin Zhou, Xin-yi Liu, Ya He, Shao-jin Zhang.

**Methodology:** Lin Zhou, Xin-yi Liu, Shao-jin Zhang.

**Project administration:** Lin Zhou, Xin-yi Liu, Shao-jin Zhang.

**Resources:** Lin Zhou, Xin-yi Liu, Shao-jin Zhang.

**Software:** Lin Zhou, Xin-yi Liu, Shao-jin Zhang.

**Supervision:** Lin Zhou, Xin-yi Liu, Shao-jin Zhang.

**Validation:** Lin Zhou, Xin-yi Liu, Shao-jin Zhang.

**Visualization:** Lin Zhou, Xin-yi Liu, Shao-jin Zhang.

**Writing – original draft:** Lin Zhou, Xin-yi Liu, Shao-jin Zhang.

**Writing – review & editing:** Lin Zhou, Xin-yi Liu, Ya He, Lu-ting Li, Shao-jin Zhang.
